# Review of recent progress toward a fiberless, whole-scalp diffuse optical tomography system

**DOI:** 10.1117/1.NPh.5.1.011012

**Published:** 2017-09-26

**Authors:** Hubin Zhao, Robert J. Cooper

**Affiliations:** University College London, Biomedical Optics Research Laboratory, Department of Medical Physics and Biomedical Engineering, London, United Kingdom

**Keywords:** diffuse optical tomography, functional near-infrared spectroscopy, fiberless, wearable, whole-scalp

## Abstract

The development of a whole-scalp, high sampling-density diffuse optical tomography (DOT) system is a critical next step in the evolution of the field of diffuse optics. To achieve this with optical fiber bundles is extremely challenging, simply because of the sheer number of bundles required, and the associated challenges of weight and ergonomics. Dispensing with optical fiber bundles and moving to head-mounted optoelectronics can potentially facilitate the advent of a new generation of wearable, whole-scalp technologies that will open up a range of new experimental and clinical applications for diffuse optical measurements. Here, we present a concise review of the significant progress that has been made toward achieving a wearable, fiberless, high-density, whole-scalp DOT system. We identify the key limitations of current technologies and discuss the possible opportunities for future development.

## 1 Introduction

The use of functional near-infrared spectroscopy (fNIRS) and diffuse optical imaging techniques has grown exponentially in recent years.^1^ However, one of the most significant challenges associated with the use of fNIRS and DOT in neuroscience and in the clinic remains the lack of a standardized, whole-scalp recording system. At present, fNIRS and DOT users must compromise on field of view,^2^ and often on both field of view and channel density.^3^^,^^4^ This has significant disadvantages. It forces researchers to design different arrays for different experimental paradigms or even subjects,^5^^–^^7^ it limits how accurately different studies can be compared and leads to significant difficulties in standardizing processing and image reconstruction methods. It also limits what can be investigated experimentally, and what can potentially be discovered, because of our inability to sample the whole cortex simultaneously.

The principal reason for the lack of a standardized whole-scalp recording system is the sheer number of sources and detectors that are required to adequately cover the adult scalp. Based upon the MNI 152 magnetic resonance imaging (MRI) atlas,^8^^,^^9^ the average adult scalp has a surface area of $∼690\;{\mathrm{cm}}^{2}$. To use a sparse 3  cm×3  cm grid layout to cover this area would require ∼38 detector and 38 source positions, significantly more than are available in most commercial fNIRS devices.^3^

While there are fiber-based fNIRS systems that have enough sources and detectors to theoretically approach sparse whole-scalp coverage (and thus allow functional measurement of the majority of the superficial cortex),^3^ for reasons of cost, and because of the mechanical difficulties associated with employing so many optical fiber bundles, it remains extremely rare for an fNIRS study (and even rarer for a DOT study) to attempt to cover the whole adult scalp.

This problem has actually become more acute in recent years, because it is now well accepted that short-separation measurements (those with a source–detector separation of less than 1.5 cm so as to principally sample the superficial tissues) are a critical component of any reliable fNIRS measurement.^10^^–^^13^ Similarly, high-density DOT measurements (those obtained using arrays with a nearest-neighbor separation of <1.5  cm) have been shown to provide significant advantages in terms of image resolution and the ability to minimize confounding signals from the scalp.^14^^,^^15^ The ideal system must, therefore, not only provide whole-scalp coverage, but do so in a high-density manner.

It is theoretically possible to achieve such a system using fiber-based methodologies. Eggebrecht et al.^2^ recently demonstrated an extended version of their high-density system that employed 188 optical fibers to cover a scalp area of $∼350\;{\mathrm{cm}}^{2}$. To cover the whole adult scalp with a comparable density would require ∼370 optode locations, which is extremely difficult to achieve using fiber optics, primarily because of their weight and relative inflexibility. The use of optical fibers to this extent also undermines some of the key advantages of DOT: namely that the technique is portable, easy to use and well-tolerated by vulnerable subjects.

Miniaturization of the optoelectronics associated with fNIRS and DOT measurements, allowing the technology to move from a bench-top to a wearable form-factor, has long been a goal of the field.^16^^–^^18^ Wearability has many potential experimental advantages, as permitting unrestricted/untethered recording, application in naturalistic environments,^19^ neurotelemetry studies^20^ and the investigation of the cerebral haemodynamic correlates of movement itself.^21^ However, the miniaturization of DOT optoelectronics also has the potential to permit the development of whole-scalp, high-channel density systems for the first time.

The principal challenges involved in the production of a fiberless, DOT system include: (1) producing a detection system with sufficient dynamic range to provide multidistance measurements (from <1.5  mm to at least 30 mm, over scalp regions where hair is present); (2) designing a system that is sufficiently high density but small and light-weight enough to allow it to be coupled directly (and comfortably) to the scalp; (3) ensuring the system can conform to the curved surface of the head; and (4) ensuring subject safety.

In this paper, we review some of the key developments that have been made toward a wearable, fiberless whole-scalp DOT system. For the sake of brevity, we have attempted to restrict this review to technologies that are continuous-wave, fiberless, and either multichannel or demonstrably expandable. Note, this review focuses solely on the developments of continuous-wave systems. A review of recent developments in time-domain DOT technology (including steps taken toward achieving a wearable device) can be found by Pifferi et al.^22^

## 2 Identified Publications

Google Scholar and Web of Science search engines were used for keyword searches: [near-infrared spectroscopy OR fNIRS OR diffuse optical tomography (DOT)] AND [wearable OR fiberless OR wireless], with the results then manual screened on the basis of the restrictions described above. We identified 17 key publications (including two presented in this current special section of Neurophotonics), details of which are summarized in Table 1. These papers include six groups of publications that describe different developmental stages of the same technology. In these cases, the quoted characteristics in Table 1 refer to the most recent available information.

**Table 1:** Characteristics of key fiberless fNIRS and DOT technologies.

Author, year	Architecture	Size of head element or module	Size of control unit	Weight	Number of source /detector locations	Demonstrated number of optical channels	Wavelengths (nm)	Demonstrated source–detector separation *in vivo* (mm)	Power consumption	Battery lifetime (h)
Kiguchi et al., 2012^24^	Control-cabled	$260\times 280\times 92\;{\mathrm{mm}}^{3}$*	$150\times 115\times 62\;{\mathrm{mm}}^{3}$*	1050 g	8/8	22	754, 830	30	0.12 mW per LD, (plus unknown control electronics)	3
Piper et al., 2013^25^	Control-cabled	12 mm diameter per optode	$103\times 43\times 167\;{\mathrm{mm}}^{3}$	1000 g (plus optodes)*	8/8	20	760, 850	25	10 mW per LED/3000 mW*	2
Sawan et al., 2013^27^	Control-cabled	11 mm diameter per optode	$160\times 130\times 82\;{\mathrm{mm}}^{3}$	800 g (plus optodes)	8/8	32	735, 850	31 (from Ref. 26)	2200 mW	24
Safaie et al., 2013^28^	Control-cabled	$80\times 35\times 10\;{\mathrm{mm}}^{3}$	—	90 g	8/4	32	760, 850	20 to 63	400 mW	5
Muehlemann et al., 2008^29^	Flex-rigid, potentially modular	$92\times 40\times 22\;{\mathrm{mm}}^{3}$	Included	40 g	4/4	16	760, 870	12.5 to 37.5	3.5/5 mW per LED, (plus unknown control electronics)	3
Hallacoglu et al., 2016^30^	Flex-rigid, potentially modular	$∼90\times 40\;{\mathrm{mm}}^{2}$	Included	—	10/18	180	690 to 850 (5 wavelengths)	∼13 to 55	—	—
von Lühmann et al., 2015^32^	Modular	$∼59\times 59\;{\mathrm{mm}}^{2}$	Included	—	4/1 per module	4	750, 850	35	11.10/10.30 mW per LED, (plus unknown control electronics)	—
Zimmermann et al., 2013^34^	Modular	$26\times 26\;{\mathrm{mm}}^{2}$	Included	—	1/1 per module	2	680, 850	20 to 50	3.2/6.3 mW per LED, (plus unknown control electronics)	—
Choi et al., 2016^35^	Modular	$200\times 200\times 80\;{\mathrm{mm}}^{3}$	Included	450 g (for prefrontal imager)	4/6 per module	14	780, 850	—	400 mA	8
Chitnis et al., 2016^37^	Modular	$30\times 30\;{\mathrm{mm}}^{2}$	Included	15 g per module	2/4 per module	128	770, 855	14 to 55	—	—
Wyser et al., 2017^38^	Modular	$25\times 22\times 10\;{\mathrm{mm}}^{3}$	Included	5 g per module	4/1 per module	4	770, 810, 850, 885	7.5 to 40	∼400 mW per module	—
Funane et al., 2017^39^	Modular	$22\times 25\times 42\;{\mathrm{mm}}^{3}$	Included	∼947 g	12/23	—	730, 855	30	320 mW per LED, 480 mW per APD	—

Note: The use of a tilde symbol (∼) indicates that these values have been estimated from details or images present in the relevant publication. The asterisk (*) symbol indicates that these values have been obtained from the corresponding commercial versions of a technology. The emdash (—) symbol indicates that the information is not available.

While the technologies described in these works vary significantly, we have attempted to group these systems into three broad categories on the basis of their system architecture. Publications^23^^–^^28^ consist of a head-mounted device on which sources and detectors are built; a standalone control module for logic operations and two-way data and power transmission; and a PC/laptop base station for data processing. Within this type of architecture, significant cabling is required for data and power transmission between the head device and the control module, and the output optical data are typically digitized a significant distance from the detectors themselves. We define this architecture as “control-cabled.” In contrast, instead of using signal cables, the fiberless system proposed in Refs. 29–31 use flexible-rigid printed circuit board (PCB) technology to connect the optical components and control unit electronics, and to locally digitize the detected optical data. Thus, we categorize these systems as “flex-rigid PCB based.” Finally, the works proposed in Refs. 32–39 employ a modular system architecture. These devices integrate the optoelectronic and control elements of their systems into head-mounted modules, which can be small in size and thus have the potential to provide excellent modularity/scalability. Throughout this paper, these devices are simply classified as “modular.”

## 3 Control-Cabled Systems

**Fig. 1:**
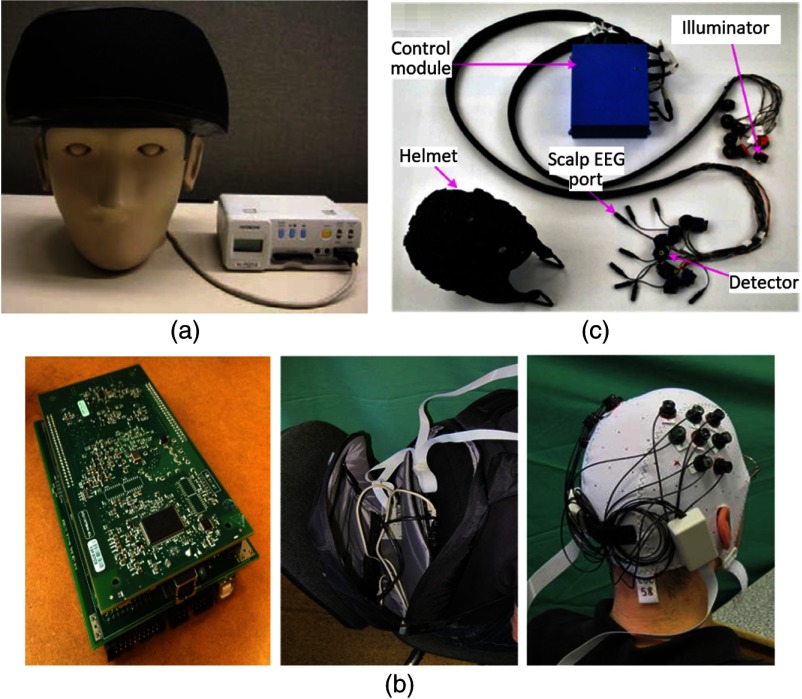
Examples of control-cabled fiberless fNIRS systems. (a) The wearable fNIRS device from Kiguchi et al., showing the headset and control unit. This figure is taken with permission from Ref. 24. (b) The wearable fNIRS system described by Piper et al., consisting of a control unit (left), a laptop base station (middle, shown stowed into a backpack along with the control unit), and a head cap in which optical components are embedded (right). This figure is taken and modified with permission from Ref. 25. (c) The wireless EEG-fNIRS system described by Sawan et al. It is comprised of a helmet (housing the optical and EEG components), cabling, and a control unit. This figure is taken and modified with permission from Ref. 27.

Atsumori et al.^23^ in 2009 and later Kiguchi et al.^24^ in 2012 proposed what they referred to as a “wearable near-infrared spectroscopy imager.” Their system consisted of three basic components: a headset, a control box, and a laptop-based control center. The prototypes of the headset and control box are shown in Fig. 1(a). The headset contained eight dual-wavelength laser diode (LD) light sources and eight avalanche photodiode (APDs) detectors and associated circuitry. A dedicated variable gain amplifier was utilized to amplify the output of each APD. In the headset, wave-guiding elements were used to guide light from the optical components to the surface of the scalp. The source–detector separation was fixed at 30 mm, and the system provided 22 measurement channels.

In this paper, six male volunteers were recruited for demonstrative *in vivo* tests. The device was successfully verified in regions of the scalp where hair is present, and the expected fNIRS activations were obtained in response to a finger tapping task. The technology described in this paper is the basis for the Hitachi WOT^40^ product series, which are commercially available.

Despite these successes, there are significant limitations to this work. First, the headset and control box are both cumbersome. The total weight of the headset is 600 g, and the weight of control box is 450 g, which will undoubtedly encumber the subject and may even cause discomfort. Furthermore, as the control module is too large to be positioned at the scalp, a signal cable must be applied to connect the headset with the control box. In addition, the use of APDs, while providing high detector sensitivity, potentially creates significant power consumption, heating, and electrical safety concerns, as each APD requires a 200-V reverse bias voltage.

Critically, this system provides a very limited number of channels, and only a single source–detector separation of 30 mm. While reducing the required dynamic range, this approach significantly limits the spatial information contained within the resulting measurements, renders the system susceptible to signal contamination from scalp haemodynamics, and makes this design inappropriate for DOT.

The system described by Piper et al. overcomes several (but not all) of the limitations of the Kiguchi system. Piper et al.^25^ employed silicon photodiode detectors in their wearable multichannel fNIRS system. Eight dual-wavelength (760 and 850 nm) light emitting diodes (LEDs) and eight silicon photodiodes were packaged into optodes and coupled to the head using a fabric cap. The packaged LEDs were placed directly in contact with the scalp, while a short length of plastic optical fiber was used to couple back-scattered light from scalp into the active area of each photodiode. Three-wire polyurethane cabling was used to link the source and detector optodes to a ribbon cable, which was, in turn, connected to a control module. The control module consisted of a data acquisition board and a custom-designed PCB, both placed within a $103\times 43\times 167\;{\mathrm{mm}}^{3}$ aluminum case. The control module was connected to a laptop via two universal serial buses for data and power transmission. The two wavelengths of each source optode were intensity-modulated at 1.0 and 1.1 kHz, with each source optode operated in turn, thus combining temporal and frequency multiplexing. The source–detector separations were configured at ∼25  mm and 20 measurement channels were created. The system overall sampling rate was 6.25 Hz. The system is shown in Fig. 1(b).

This system was validated on eight subjects, and it was the first fNIRS system to be demonstrated *in vivo* during outdoor activity (in this case, cycling). The fabric cap provided a comfortable interface, and the relatively compact design of the control module facilitated the wearing of the system in a backpack. Compared to the work described by Kiguchi et al.,^24^ the adoption of silicon photodiodes improved power consumption, and likely the ease with which the system can be rendered safe for use. However, much like Kiguchi et al., the overall weight and size of this system are still limiting. This system is likely too large to facilitate comfortable application to children or infants. Furthermore, significant cabling is required for data and power transmission between the optical components at the scalp and the control module, adding a further burden of weight for subjects and rendering the system susceptible to radio frequency (RF) noise. Extra cabling is also likely to increase susceptibility to motion artifacts. Critically, the dynamic range of this system is not defined, and the demonstrated range of source–detector separations in this paper is very narrow. This technology gave rise to the NIRx NIRSport commercial fNIRS device.^41^ The commercial version of the system can provide 16 sources and 16 detectors, but with commensurate increases in size and weight.

In 2011, Lareau et al.^26^ described a wearable system to achieve simultaneous fNIRS and scalp electroencephalography (EEG). Building upon this, Sawan et al. upgraded the technology to yield a more advanced wireless system,^27^ which is shown in Fig. 1(c). This system consisted of two major elements: a helmet made of flexible neoprene and a control module. The helmet was equipped with eight dual-wavelength (735 and 850 nm) LEDs and eight APDs, in addition to eight EEG electrodes. The LEDs were time-multiplexed, and each LED was modulated at 1 kHz. The APDs were employed to ensure satisfactory sensitivity when a −150-V bias voltage was applied. Each source could be coupled with 4 detectors, thus up to 32 optical channels could be generated. The LED driving circuitry and the detection circuitry were compressed into circular PCBs, each with a diameter of 11 mm. Once again, multiple cables were used to connect the source and detector components to a distant control module, for data and power transmission. However, the inclusion of a Bluetooth module provided the significant advantage of allowing data to be transmitted wirelessly from the control module to a base laptop.

However, once again, this system includes high-bias voltage APDs, extensive cabling, and a large control module ($160\times 130\times 82\;{\mathrm{mm}}^{3}$), which cannot be directly positioned at the scalp. Moreover, the dynamic range and achievable source–detector separations are not specified in this paper. As such, it is impossible to assess the suitability of this technology for expansion to sample the whole scalp or facilitate DOT.

Similar work was conducted by Safaie et al.,^28^ in 2013, which aimed to develop an integrated, wireless EEG-fNIRS system. As per Refs. 23–27, this system was comprised of three basic components: the head-mounted optoelectronics (in this case eight dual-wavelength LEDs and four silicon photodiodes), a control module, and a laptop. In this system, the LEDs were time-multiplexed, and the system was designed to provide up to 32 channels with source–detector separations potentially ranging from 20 to 63 mm. Note, however, that the authors provide no discussion of data quality at longer separations, and while a theoretical effective dynamic range of 198 dB is discussed, the practical dynamic range (which will be largely a function of detector dark-current noise) is not demonstrated. A Bluetooth module was incorporated to realize wireless data transmission between the control module and laptop. The optoelectronic “patch” measured $35\times 80\times 10\;{\mathrm{mm}}^{3}$ and weighed only 90 g. The system exhibited a total power consumption of 400 mW.

This system achieved significant advances over Refs. 23–27. It demonstrated wireless data transmission, with a relatively small and lightweight control module, employed a safe supply voltage, and demonstrated low power consumption. However, all these devices have common disadvantages, including the use of extensive cabling (specifically cabling between the optical components and the control module) and an architecture that limits the expansion of these technologies to sample the whole scalp. The technologies described in Refs. 25–28 also require analog signal transmission from the detectors to the distant control module, potentially leaving these systems vulnerable to RF noise.

Note there are also several commercially available wearable systems that are not described here. These include the Brite 23 (Artinis Medical Systems, The Netherlands),^42^ the Genie (MRRA),^43^ and systems from fNIRS Devices,^44^ Spectratech Inc., Japan,^45^^–^^47^ and Shimadzu Corp., Japan.^48^ These devices are not discussed here because they either do not meet our review criteria or have not been described sufficiently in peer-reviewed publications.

## 4 Flex-Rigid Printed Circuit Board-Based Technologies

**Fig. 2:**
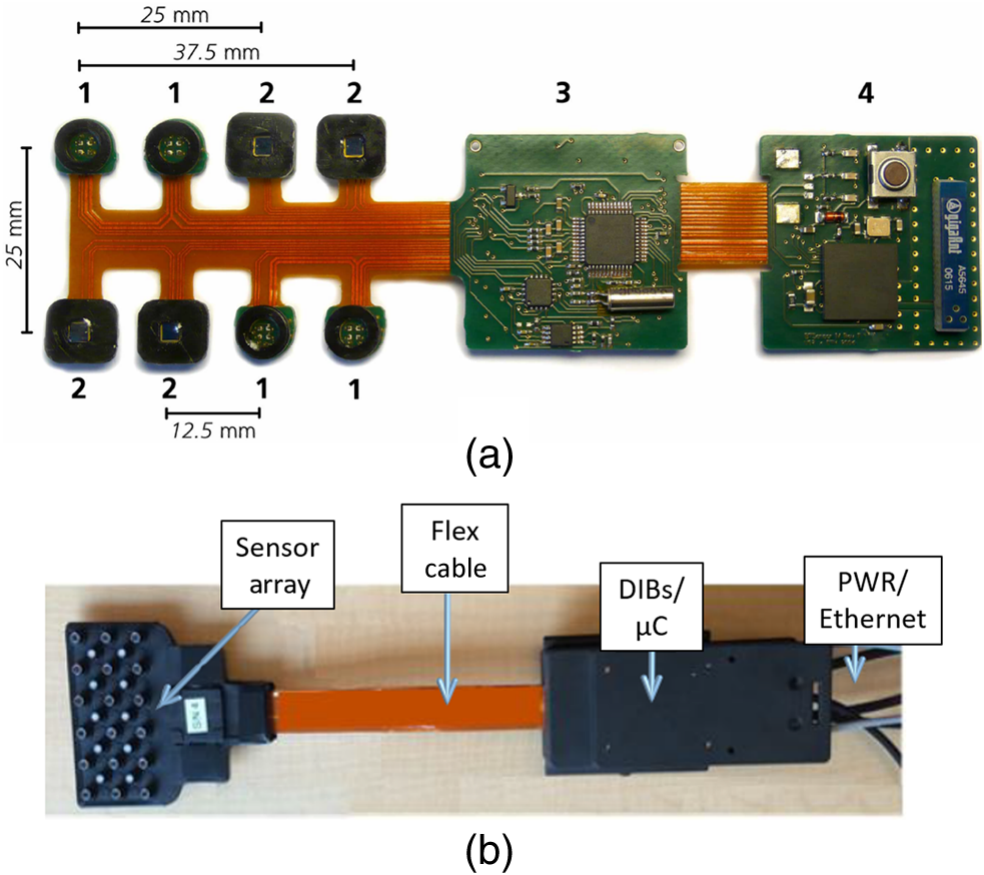
Examples of flexible-rigid PCB-based technologies. (a) The miniaturized wireless fNIRS system developed by Muehlemann et al. The LED sources (labeled 1) and PIN photodiode detectors (labeled 2) are connected to control electronics using flexible PCB; control and power electronics (labeled 3) and Bluetooth module (labeled 4) are embedded on two separated rigid sections. This figure is taken with permission from Ref. 29. (b) The compact flex-rigid PCB-based DOT system described by Hallacoglu et al., which consists of a high-density sensor array, a flex cable, and DIBs/microcontroller (μC) section with power (PWR) and Ethernet connections. This figure is taken and modified with permission from Ref. 30.

In 2008, Muehlemann et al.^29^ were the first to take a different approach: utilizing a flex-rigid PCB technology to accomplish a miniaturized wireless fNIRS system [Fig. 2(a)]. The device consisted of four parts: light sources; detectors; drive and detection electronics and power supply; and wireless communication components. This device contained four dual-wavelength LED light sources and four PIN photodiode detectors. The LEDs were time-multiplexed with a 120  μs on period per sample. The source–detector separations were configured at 12.5, 25, and 37.5 mm, and 16 channels were produced.

The LED drivers were embedded on a rigid part of the PCB, along with power management circuitry and a 12-bit analog-to-digital converter (ADC). On a separate rigid section, a Bluetooth communication module was included to transmit the digitalized optical intensity data to a host PC/base station. The flexible section was encased within medical grade silicone, to ensure patient comfort and safety. The full device dimensions were $92\times 40\times 22\;{\mathrm{mm}}^{3}$, and it weighed just 40 g.

Because of its relatively small dimensions, its low weight, and cableless construction, this device can be placed directly at the scalp, ensuring wearability. The flex-rigid construction is critical to this design because it allows the optical components to conform to the curved subject scalp while being directly connected to the support electronics without additional cabling. Unlike the systems described in Refs. 25–28, this system digitizes the recorded optical intensity data very close to the detector itself, which will help minimize noise.

However, this system is not without limitations. One significant concern is dynamic range, which will be limited by the choice of a 12-bit ADC and a low-performance microcontroller. However, this publication is from 2008, and these issues could now be improved by simply upgrading both the ADC and microcontroller unit (MCU).

In theory, this device could be expanded or augmented to cover the whole adult scalp. However, the support electronics and wireless communication modules currently occupy a significant area, which would, therefore, not be sampled by optical sources and detectors. To expand this device, or develop source encoding and communication protocols sufficient to allow the simultaneous operation of multiple independent devices, would likely require a complete redesign of the technology. Furthermore, how mechanically robust the device will remain after repeated bending cycles of the flexible PCB is unclear.

In 2016, Hallacoglu et al. described a significantly more advanced flex-rigid PCB-based system that is more compact and (critically) was one of the first fiberless systems to incorporate high-density measurements.^30^^,^^31^ This system, which is under commercial development for clinical applications by Cephalogics,^49^ consists of three main parts: a high-density sensor array, a flexible cable, and a digital interface board (DIB)/microcontroller, [Fig. 2(b)]. The sensor array consists of 10 source positions and 18 detectors. Each source consists of five time-encoded, amplitude-modulated vertical cavity surface emitting lasers (VCSELs), at wavelengths ranging from 690 to 850 nm. The system employs silicon photodiodes as detectors. The available source–detector separations theoretically range from 13 to 87 mm over nine nearest-neighbors. As a result, up to 180 measurement channels can potentially be produced at each wavelength over an area of $∼90\times 40\;{\mathrm{mm}}^{2}$. Flexible cabling is employed to connect the sensor array and the DIBs/microcontroller to achieve data and power transmission, and an Ethernet connection was built to accomplish two-way communication between the proposed DOT system and base station/laptop.

This high-density DOT system has been verified on both blood phantoms and human subjects. The sensitivity and dynamic range of this system are very impressive. Measurements of the human brain, over regions of the scalp where hair was present, were achieved for source–detector separations ranging from 13 to ∼55  mm. While this range is smaller than that implied by the claim of a 140 dB of dynamic range, this range is significantly larger than is common in commercial benchtop fNIRS technologies.^3^ However, the use of a very large number of laser sources may potentially cause issues with power consumption and heat generation, and like the device developed by Muehlemann et al., it is not clear how easy it would be to apply more than one of these devices simultaneously to the same subject. As yet, no attempt has been made to make this device wireless. While wireless operation may not be necessary for clinical use (which appears to be the principal target application), the large flexible cable would become an issue in the expansion of this technology to sample the whole scalp.

## 5 Modular Devices

An alternative approach to individually cabled optodes, or flex-rigid circuitry, that can still allow the points of optical contact to conform to the head is to pursue a modular system architecture. Developing a module that contains source and detector optics, dedicated control electronics, is small enough to allow the optical contacts to conform to the curved scalp and can be used in conjunction with multiple other modules has numerous advantages.

**Fig. 3:**
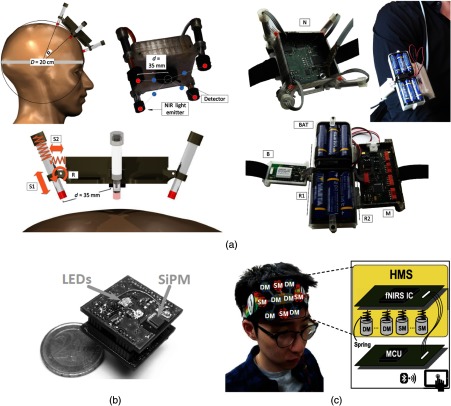
Examples of modular devices. (a) The spring-loaded modular fNIRS device described by von Lühmann et al. The subfigure on the left demonstrates the mechanical spring-loaded concept; the subfigure on the right shows a single fNIRS module and the base station: N represents the fNIRS module, B indicates the Bluetooth module, BAT indicates the battery, R1/R2 are the rotatory joints, and M indicates the main aboard. This figure is taken with permission from Ref. 32. (b) The SiPM-based modular fNIRS device from Zimmermann et al., constructed on two stacked PCBs. The upper PCB houses the optoelectronics, while the lower board acts as a control unit. This figure is taken with permission from Ref. 34. (c) An IC-based modular fNIRS device described by Choi et al., consisting of a head-based subassembly and an external MCU. This figure is taken with permission from Ref. 35.

In 2015, von Lühmann et al.^32^ proposed a modular system architecture to accomplish a multichannel fNIRS system. The system principally consisted of two elements: an fNIRS module and a base station, as demonstrated in Fig. 3(a). In the fNIRS module, four dual-wavelength LEDs (750 and 850 nm) were perpendicularly fixed at the corners of the module to function as source locations, while a single silicon photodiode was placed at the center to act as detector. The sources were time multiplexed. The source–detector separation was fixed at 35 mm, and four optical channels were generated within each module. The associated LED driver, detection electronics, and local logic controller all formed part of the fNIRS module. An 8-bit digital-to-analog converter (DAC) was employed to modulate the LED drive current in 256 levels. An 8-bit microcontroller, local to each module, was adopted to generate predefined reference signals and also to control logic timing. Thus, the fNIRS module combined the optical sources and detectors, and the control electronics into a single unit.

The base station contained a 16-bit bit ADC, a more powerful microcontroller for data acquisition and module control and a Bluetooth module for wireless data transmission. The overall system dynamic range was 55.13 dB. The system can potentially be scaled up to include four modules, and the authors claim up to 16 fNIRS channels could be generated. This implies, however, that optical channels cannot be generated across modules, which is potentially a significant limitation. Note that this system has been improved further through an upgrade of the ADC to 24 bit and the inclusion of accelerometry.^33^

Despite the many merits of this system, there are still several critical limitations. First, the fNIRS modules themselves are very large, and a complex articulated mechanical system is required to ensure the sources can make contact with the scalp. The size of the modules is a limitation not simply because of the mechanical implications, but because any array resulting from the application of multiple modules (which only contain 35-mm channels) will intrinsically provide only sparse spatial sampling. The weight of the modules is not stated.

In 2013, Zimmermann et al.^34^ described another modular fNIRS technology. In this work, commercially available silicon photomultipliers (SiPM) were utilized as detectors in a miniaturized fNIRS instrument. The proposed modules were constructed using two stacked PCB boards, as displayed in Fig. 3(b). The upper PCB board housed the optical sources and detectors along with associated circuits. The lower PCB served as a control module, in which a microcontroller and data acquisition components were embedded. In each module, two LEDs (680 and 850 nm) were adopted as the source while one SiPM was used as the detector. The LEDs were time-multiplexed with a 33.3% duty cycle. The SiPM was configured with a tuneable bias voltage (varied between −32 and −24  V), allowing the detector gain to become programmable. Two of these modules were demonstrated, and the system allowed either module to act as source or detector at any given time, thus allowing channels to be formed across modules. The modules were $26\times 26\;{\mathrm{mm}}^{2}$ in area, and the system provided an overall sampling rate of 100 Hz. Source–detector separations of between 20 and 50 mm were obtained *in vivo* on a subject’s arm.

The fNIRS module demonstrated by Zimmermann et al. is outstandingly compact and provides the possibility of producing multiple modules to construct a multichannel fNIRS system. However, while the scalability of this modular system is a clear goal, it would undoubtedly be a significant challenge to integrate many modules into a high-density DOT system and cover the whole scalp, and these challenges have yet to be addressed. Note that this work was the first to demonstrate the feasibility of utilizing SiPM detectors in fNIRS systems. Employing SiPMs potentially has several advantages over silicon photodiodes and APDs: they provide a higher sensitivity than silicon photodiodes but are similarly small, lightweight, and durable. They require a negative bias voltage of 24 to 32 V, which is far lower than APDs, but still high enough to require careful design of the system’s power distribution to ensure safety and efficiency.

In 2016, Choi et al.^35^ described a device based on a custom-designed integrated-circuit (IC). In this system, there were two major elements: a head-mounted subassembly and an external MCU, as shown in Fig. 3(c). The IC design was embedded into the head-mounted subassembly, along with the source and detector modules. The IC provided source control, data filtering, quantization, and serialization. A Walsh-code generator and source drivers were developed for source encoding, and an 8-bit DAC was used to adjust the driving current. On the detector side, two programmable gain amplifiers and a variable-gain operational trans-conductance amplifier were developed along with a 12-bit ADC for optical data acquisition and conversion. All these functional circuits were packaged into a single IC, which provides eight parallel source chains and ten parallel detector chains. The IC was connected to four dual-wavelength VCSEL sources (780 and 850 nm) and six silicon photodiode detectors. The system demonstrated outstanding sensitivity, with a noise-equivalent power of only 400 fW.

Metal springs were adopted to mechanically connect all sources and detectors to the IC package and allow the optical contacts to conform to the scalp. Fourteen measurement channels were produced in a single-head-mounted subassembly. Up to three subassembly modules can potentially be employed simultaneously, providing up to 42 measurement channels. The external MCU was used for logic control, and data and power transmission. A Bluetooth module was integrated to accomplish wireless data transmission between the head-mounted system and a PC/tablet computer. The overall sampling rate was variable between 7.8 and 31.25 Hz, and the total current consumption was 400 mA. The proposed multiunit system measured $200\times 200\times 80\;{\mathrm{mm}}^{3}$, with a total weight of 450 g.

This work proposed a distinct approach to develop a wearable diffuse optical device: using a compact IC design. The overall system size and weight are impressive, and the control electronics are positioned directly at the scalp. However, from this sole paper, it is unclear whether multiple source–detector separations can be employed, and the choice of source–detector separations in the demonstrated systems is not specified. It is, therefore, difficult to comment on the system’s potential sampling density, its dynamic range or its suitability for high-density measurements and DOT. Critically, this system has been demonstrated only over the forehead, and it is unknown whether this system can be applied at other areas of interest. Furthermore, while this system can provide 42 channels, Choi et al. have yet to attempt to address the challenges associated with expanding this technology to provide whole-scalp sampling. This technology formed the basis of the Obelab NIRSIT system,^50^ which provides prefrontal cortical sampling and is now commercially available.

**Fig. 4:**
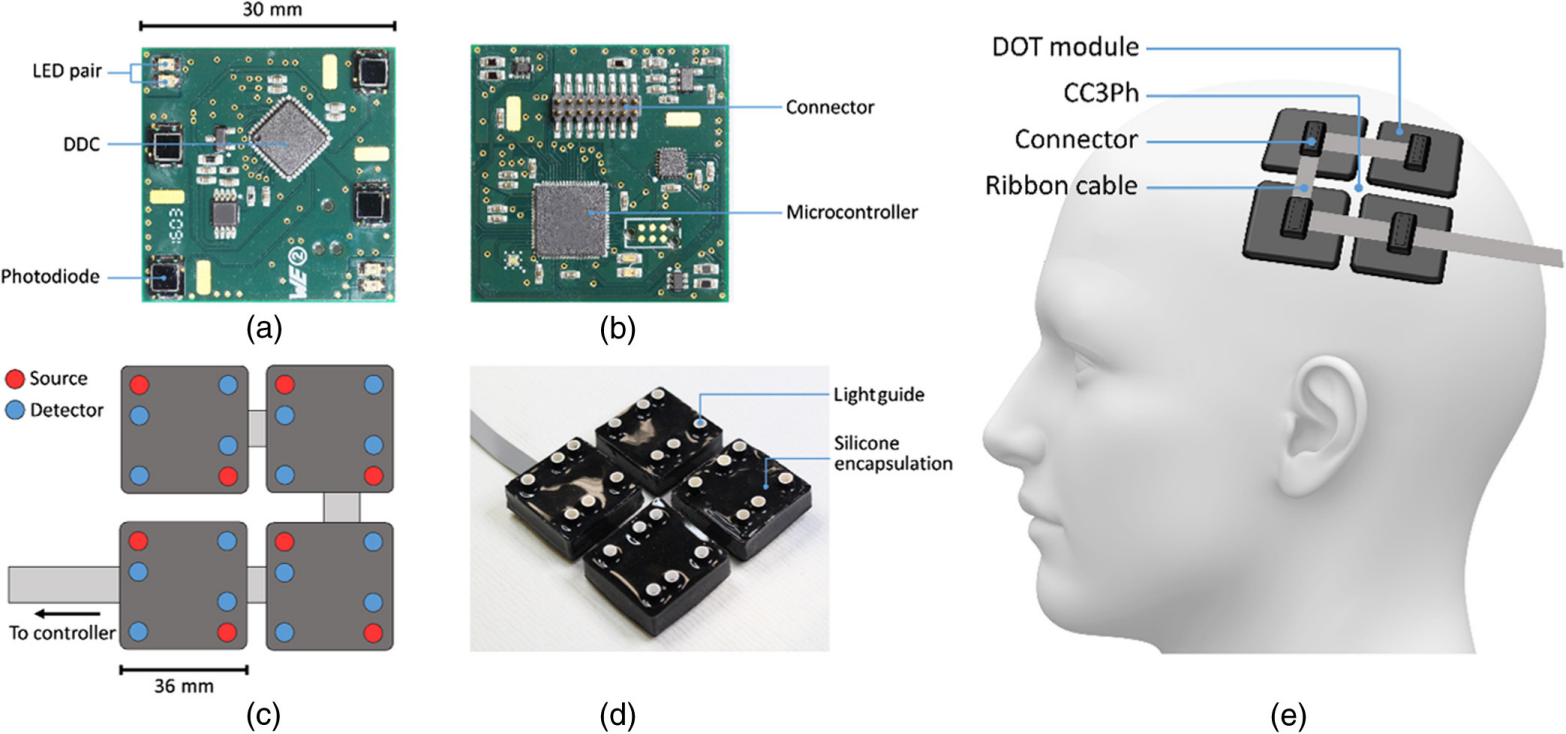
The modular DOT system described by Chitnis et al. The front and rear views of the modular PCB are shown in (a) and (b), respectively. The 2×2 grid pattern of the system is shown in (c), and the encapsulated system is shown in (d). (e) Demonstrates how this four-module system was arranged on the scalp. This figure is taken and modified with permission from Ref. 37.

Also in 2016, Chitnis et al. demonstrated a fiberless, multiwavelength fNIRS system, designed to allow multiple chromophore measurement.^36^ Eight-wavelength optical sensing was achieved, with an 80-dB dynamic range. Building on this technology, Chitnis et al. have subsequently described a fiberless, high-density DOT system^37^ (Fig. 4) that consists of multiple-independent DOT modules.

A compact PCB (30  mm×30  mm) was fabricated to house two dual-wavelength LED sources (at 770 and 855 nm) and four silicon photodiodes. Each photodiode was connected to a high-resolution charge-to-digital converter, which included an integration amplifier and a 20-bit Sigma-Delta ADC. This amplification strategy provided a demonstrated dynamic range of 98 dB and a noise-equivalent power of just 370 fW. A local microcontroller was embedded within each module for logic control and data processing, and the sources were time-encoded.

A custom-designed, multilayered silicone encapsulation was adopted to ensure module integrity and subject comfort. Each module weighed only 15 g. Critically, the system required only a single multistrand ribbon cable to connect the modules to one-another, and to a base unit, which allowed multiple modules to be arbitrarily arranged on the scalp. In the reported work, four modules were employed and configured in a 2×2 grid pattern that provided up to 128 measurement channels (per wavelength) and theoretical source–detector separations ranging from 8.5 to 85 mm. In a study of six subjects undertaking a motor task, good-quality data were obtained in channels with source–detector separations ranging from 14 to 55 mm, in the presence of hair.

This technology has a number of key features that are likely to be critical to the development of a fiberless, whole-scalp DOT system. These include a high-density sampling, a high dynamic range, field-leading sensitivity, modules that are sufficiently small to conform to the scalp, minimal cabling and, critically, scalability. The authors report that their system can theoretically support up to 75 modules, more than would be required to cover the whole adult scalp. However, this has yet to be demonstrated, and there remain a number of challenges that must be overcome before this goal can be achieved. Currently, a single cable is used for data transmission, and the subject, therefore, remains tethered. However, the authors state that standard WiFi or Bluetooth protocols would readily allow wireless data transmission. To date, Chitnis et al. have demonstrated the simultaneous use of only four modules. Depending on the intermodule spacing, 45 to 55 modules would likely be required to cover the full adult scalp. It remains to be seen whether this is achievable. The modules demonstrated by Chitnis et al. also exhibited an issue with the regular saturation of their shortest-separation channels, and (because of the use of time-encoding) exhibited a relatively low sample rate of 2.9 Hz, an issue that will likely become more challenging as the number of modules increases.

In this current special section of neurophotonics, Funane et al.^39^ described a development of their previous cable-controlled system^24^ (see above) that uses a modular design structure to once again achieve an fNIRS system that covers the forehead. This device consists of separate source and detector modules but adopts a serialized communication architecture similar to that demonstrated by Chitnis et al.^37^ The authors described a system consisting of 12 LED modules and 23 APDs modules, with a fixed source–detector separation of 30 mm, which can potentially be arranged to form a variety of NIRS arrays.

Also in this special section, Wyser et al.^38^ demonstrated an advancement of Zimmermann’s work,^34^ which consists of a redesigned module with a small ($∼22\times 25\;{\mathrm{mm}}^{2}$) hexagonal form factor and a serialized communication architecture as per Chitnis et al.^37^ The design maintains a SiPM detector, similar to that described by Zimmermann et al., but now includes four LEDs (770, 810, 850, and 885 nm) to provide a source within each module. By upgrading to four wavelengths, Wyser et al.^38^ show the potential for measuring concentration changes in multiple chromophores.^36^ The authors demonstrated a four-channel system created from the simultaneous application of two modules, but discussed the potential extension of their design to include multiple modules and sample extended regions of the cerebral cortex.

## 6 Discussion and Conclusion

In recent years, anatomical modeling and meshing procedures, and the use of registered MRI data and generic atlases have been shown to be an effective solution to the problem that diffuse optical methods do not intrinsically provide structural information.^5^^,^^51^^,^^52^ Meanwhile, the use of high-density measurements and tomographic image reconstruction has also been extensively demonstrated to provide improved data quality, resolution, and depth specificity.^53^^–^^55^ However, despite the repeated demonstration of the utility of these methodological advances, they are still unavailable to most users. One major reason for this is because these processes have yet to be standardized, automated, and rendered user-friendly.

A significant barrier to automation and standardization of fNIRS data processing and diffuse optical image reconstruction is the lack of hardware that allows users to consistently apply sources and detectors across the whole scalp, and sample the majority of the superficial cortex uniformly in all subjects. While there are many commercially available systems that provide upward of 20 sources and detectors, this is still far too few to cover the adult scalp in anything other than a highly sparse arrangement. As a result, users are still forced to find a balance between sampling density and field of view.^3^

Removing the burden of optical fiber bundles is critical to the development of the fNIRS field and to the acceptance and uptake of DOT across neuroscience and clinical neurology. As we have shown here, a number of key steps have been made toward this goal in recent years. The first was the development of low channel-count, fiberless fNIRS systems as described by Kiguchi et al.,^24^^,^^23^ and Piper et al.^25^ Such devices have been commercially available for several years, but do not provide a sufficient number of channels to allow uniform whole-scalp sampling, even in a highly sparse manner. Furthermore, while the forehead-mounted systems^23^^,^^24^^,^^35^^,^^39^ and the cumbersome control-cabled designs^25^^–^^28^ can untether the subject from the laboratory bench, they do little to improve the ergonomics of diffuse optical imaging devices beyond what can already be achieved with optical fiber bundles.

The use of flexible PCBs is a promising avenue of investigation, as they make it possible to construct devices with excellent sampling density and locally positioned support electronics that can still conform to the curves of the scalp.^29^^,^^30^^,^^31^ However, the mechanical characteristics of flex-rigid PCB are likely to limit just how well these devices can conform, as the material cannot simultaneously conform in two dimensions. Furthermore, it is not apparent whether the devices described by Muehlemann and by Hallacoglu can be expanded, or whether multiple such devices can be used simultaneously to improve the field of view.

Modular designs, in which each module is small enough to allow its optical contacts to conform to the scalp, appear to be the single most promising approach for achieving a wearable, high-sampling density, whole-scalp diffuse optical imaging system. Designs such as that described by Zimmermann and that described by Chitnis provide local signal digitization and a high dynamic range. They also achieve both intra- and intermodule channels, providing a large variation of source–detector separations, which is critical in an effective DOT system. Optimization of the layout of sources and detectors both within and between modules will be key to future development of modular fiberless DOT devices.

Chitnis et al. have already demonstrated the ability of their system to obtain high-density DOT measurements of the adult human brain. However, there remain several fundamental challenges that have yet to be addressed. One is simply ergonomics: i.e., how to place wearable optodes across the whole scalp in a high-density pattern and at the same time ensure satisfactory comfort for subjects and patients. Any head-mounted device must be lightweight, flexible, and robust. Achieving good optical coupling in the presence of hair is also a critical challenge. In traditional fNIRS systems, any hair beneath an optical fiber can usually be manually moved aside to clear the optical path. However, wearable fiberless devices have the potential to accommodate many more sources and detectors, making it impractical to manually move the hair aside from under each optical connection. Furthermore, wearable components and electronics can potentially physically prevent users having access to the scalp, adding more complexity to the process of optimizing optical coupling.

To overcome these challenges will require a multidisciplinary effort encompassing optoelectronic and mechanical design and will require the sourcing of appropriate biocompatible materials, precision fabrication, and encapsulation. Another key challenge for multichannel, high-density systems will be found in high-speed data communication and processing. Sophisticated control electronics and data interfacing methods will be necessary to ensure high-speed, high-resolution, large-dynamic range data acquisition. This will result in significant additional complexity in both hardware and software.

Safety is also a key concern. Electrical safety is clearly critical, and operating within a safe voltage range is an advantage. There are also potential safety issues related to heat dissipation: the high power consumption of any system that incorporates a very large number of optical sources will potentially cause issues of localized heating. Furthermore, providing sufficient battery life for stable experimental application will be a challenge. Ultralow-power electronic designs, and more efficient data acquisition sequences, will likely be necessary to minimize heating and increase the battery lifetime for long-term operation.

The appropriate choice of source and detector technologies is clearly vital. On the source side, LDs have the advantage of providing a collimated output with a narrow bandwidth, but they cannot be placed directly at the scalp, because of concerns regarding heating. The use of LEDs avoids many of the safety challenges associated with LDs, but LEDs typically have a wide emission angle (which often results in inefficient optical coupling) and a relatively wide bandwidth. Recently, VCSELs have been successfully demonstrated in several fNIRS/DOT systems.^30^^,^^35^ VCSELs have the advantage of high efficiency, low power consumption, and narrow bandwidth,^56^^,^^57^ and are also compatible with conventional fabrication processes. However, as an emerging technology, prepackaged VCSELs with appropriate wavelengths, optical power, and package size remain relatively rare.

On the detector side, the noise equivalent power (NEP) is likely the single most important factor. However, as NEP is a combination of gain and noise characteristics, it is dependent on multiple factors including bias voltage, gain, active area, system sampling rate. It is, therefore, difficult to make any general judgment about the choice of appropriate detector technology for fiberless DOT systems. The high-gain APDs that are common to fiber-based fNIRS systems are likely to be inappropriate for whole-scalp wearable technologies because of their high-voltage operation. Like APDs, SiPMs also have high gain, but require lower bias voltages (24 to 32 V). Both APDs and SiPMs are usually subject to significant dark current, which can increase the NEP. Photodiodes operate at low voltages, are inexpensive and widely available.

In this paper, we have sought to highlight the significant progress that has been made toward a wearable, whole-scalp, high-density DOT technology. In the process of compiling this review, the authors have noted significant variation in the manner that the performance of fNIRS and DOT devices are described in peer-review publications. It is surprisingly common for key characteristics of these devices to be omitted from publication altogether. In an attempt to address this issue, we propose the following list of key characteristics, which we believe constitutes a reasonable minimum expectation for the peer-reviewed description of any wearable fNIRS or DOT device:

• Number of source positions
• Number of detector positions
• Source wavelengths and source bandwidths (nm)
• Source output powers (Watts)
  – A measured value taken directly from the device so as to account for any light-guide or coupling components.
• Source encoding strategy and approach to background light
  – e.g., Spatial, temporal, frequency.
• Noise-equivalent power (Watts)
  – A measured value obtained in a manner consistent with standard operation of the device.
• Maximum measureable power (Watts)
• Dynamic range (dB)
  – A measured value obtained in a manner consistent with standard operation of the device.
• Maximum and minimum achievable source–detector separations (mm)
  – The range of source–detector separations for which the measured power exceeds the noise-equivalent power and remains beneath the maximum measurable power, as measured during standard operation of the device, ideally when applied to adult subjects with hair.
• Maximum achievable channel density (number of channels $\mathrm{per}\;{\mathrm{cm}}^{2}$)
  – As calculated using the total number of channels that fall within the achievable range described above.
• Full frame rate (Hz)
  – The inverse of the time taken to sample all channels that fall within the achievable range described above.
• Power consumption (Watts)
• Battery lifetime (Hours)
• Weight (g)
• Dimensions (mm)
• Conformability
  – i.e., The systems’ ability to conform to the curved scalp should be described.
• Subject comfort
  – e.g., The duration of the device can be worn without reported discomfort.
• Safety considerations
  – e.g., Operating voltage of on-head elements, heating effects.

Within the next few years, it seems highly likely that wearable, fiberless, high-density, and whole-scalp diffuse optical imaging technologies will become readily available. The advent of these technologies will promote standardization and automation of image reconstruction and DOT data processing methodologies, will enable whole-scalp functional imaging of the human brain in almost any environment, and will dramatically accelerate the growth of diffuse optical imaging throughout neuroscience and clinical neurology.
